# Simulation Analysis and Experimental Study of the Strength of Aluminum Alloy Suspension Structure

**DOI:** 10.3390/ma16010128

**Published:** 2022-12-23

**Authors:** Wenxue Qian, Ningxiang Wu, Hao Li, Xiaowei Yin, Liyang Xie

**Affiliations:** 1 School of Mechanical Engineering and Automation, Northeastern University, Shenyang 110819, China; 2 Key Laboratory of Vibration and Control of Aero-Propulsion Systems, Ministry of Education, Northeastern University, Shenyang 110819, China; 3 School of Mechanical, Shenyang Institute of Engineering, Shenyang 110136, China

**Keywords:** aluminum suspension structure, simulation analysis, fatigue life, load–life curve

## Abstract

High-speed trains have a large amount of ancillary equipment, which is suspended from the underside of the train by means of a suspension structure. Due to the large mass of the ancillary equipment, the suspension structure is subjected to various loads during train operation and there is a risk of fatigue failure. In this paper, the stress distribution at the suspension point and the lo-cation of the maximum stress point under load are investigated in detail based on actual test loads at the suspension point and finite element simulation analysis. In order to further investigate the fracture failure of the suspension points, experimental studies were carried out. Firstly, static strength tests were carried out to obtain the load–displacement curves of the structural members and to determine the fracture strength of the structure based on the displacement sensors, and secondly, fatigue tests at different stress levels were carried out to obtain the load–life curves of the structural members and to investigate the probabilistic load–life curves at different reliability levels. The test results show that the structural component has a high fracture strength of 65kN, while the conditional fatigue strength is relatively low, corresponding to a load level of 12.5kN at a median life of 10^6^ cycles. The above research work provides the necessary basis for the design, optimization and reliability assessment of the suspension structures of high-speed trains.

## 1. Introduction

High-speed trains (HST) have become one of the most important modes of transport in the world because of their high passenger capacity, high transport capacity, safety and reliability and high speed [1]. More and more countries are building and planning to build high-speed rail lines. With the continuous increase in ownership, energy saving and environmental protection have also become important indicators in the design of HSTs. The lightweight design of HSTs is an effective means to achieve energy saving and environmental protection [2,3].

Aluminum alloys are increasingly being used in the construction of high-speed trains due to their low density, high strength, good thermal conductivity, ease of forming and aesthetically pleasing construction [4,5,6,7,8]. Aluminum is commonly used in the manufacture of structural components for high-speed trains, such as brackets, special frames, underframes and side beam structures [9,10]. One of the main functions of the base plate is to carry various train equipment, such as traction transformers, traction converters, battery boxes, inverter boxes, cooling units, etc. [11]. These are large pieces of equipment that are suspended in slots in the base plate structure by means of several suspension bolts [12]. The bolts are usually high-strength bolts made of alloy steel, which have high static and fatigue strengths, and the individual bolts are not subjected to very high forces due to the sharing of the load by several bolts [13]. However, for suspension slots, the thickness of the slots is usually small due to the lightweight requirements. In service, high-speed trains experience a variety of different railway conditions and environments, resulting in the train body structure being subjected to a variety of different loads and the possibility of failure under the long-term effects of various variable loads [14,15,16,17]. A range of research work has been carried out on the static and fatigue strength failures of aluminum alloy structures. Zhou et al. [18] studied the aerodynamic fatigue of train equipment cabins. An eight-level load spectrum was constructed by means of rainflow counting and studied using Miner’s rule and non-destructive testing. The results confirmed that the welds played a detrimental role rather than the base metal, and that more damage was caused to the base metal than the welds when the train passed through the tunnel. Lu et al. [19] investigated in detail the effect of vibration modes on the fatigue damage of bogies for high-speed trains under random loading conditions, considering an extended range of excitation frequencies and an increased proportion of high-frequency components. Firstly, a detailed finite element model of the bogie was developed and the restrained modes were analyzed using the Block Lanczos modal analysis program. Next, a full-scale rigid–flexible coupled vehicle dynamics model was developed to explore the maximum fatigue damage at hot spot locations influenced by the mode. The results show that the modal superposition method based on the dynamic design approach is a very effective method for investigating vibration-induced fatigue strength problems. By improving the dynamic performance of the bogie, the fatigue reliability of the bogie can be improved. Hu et al. [20] carried out a systematic study of fracture analysis, finite element analysis and field tests under actual stress and acceleration conditions on the cracking of a high-speed train gearbox housing. The time, frequency and time–frequency domain analyses showed that the first two natural modes of vibration were excited by the irregularities of the out-of-round wheel and track, and that the superposition of these modes of vibration led to the onset of the beat phenomenon, where the amplitude of the vibration modulates periodically, which increases the likelihood of exceeding the fatigue limit and generating greater structural damage. The analysis shows that the fatigue strength in the failure region is reduced by the casting process, which ultimately leads to the formation of fatigue cracks. Guo et al. [21] proposed a new method for assessing the life of a power bogie frame considering internal excitations. To achieve this, a modified torsional vibration model that takes into account the gear meshing is first developed. The results of the calculations are then fed into a rigid–flexible coupled vehicle dynamic model to estimate the dynamic response and thus determine the cumulative damage. Validation results indicate that internal excitation should be considered to determine the service life. Yu et al. [22] proposed and compared two robust design methods for aluminum alloy sidewall fixtures for high-speed trains based on the Taguchi method and the dual response surface method. Based on the simplified shell finite element model of the sidewall and the welding deformation evaluation index of the sidewall, the original scheme, the fixture design scheme based on the Taguchi method and the fixture design scheme based on the dual response surface method were compared, indicating that the sidewall fixture design method based on the response surface method is more suitable for the robust fixture design of the sidewall. The proposed robust fixture design method can also be applied to other similar problems.

Fatigue failure is the most important form of failure of aluminum alloy structural components for high-speed trains. A great deal of work has been conducted by scholars on material properties, load forms and fatigue life prediction methods, but due to the complexity of fatigue failure, many problems have not been completely solved [23,24,25,26]. Khan et al. [27] examined cracks in Al2024 T351 specimens subjected to low cyclic fatigue loading by means of a non-breaking X-ray inspection technique. Based on data reconstruction, the three-dimensional image visualization of the internal structure was achieved, verifying the common assumption that macroscopic cracks form on the surface of high-strength aluminum alloy Al2024 under fatigue and begin to extend internally during monotonic loading, and also uncovering new findings relating to the dependence of triaxiality on the resulting fracture process, as well as those relating to the onset of damage caused by annealing. Wu et al. [28] investigated the mechanical properties of pultruded aluminum truss-core sandwich panels. The bending performance and damage pattern of two aluminum material joist-core sandwich panels were investigated by three-point bending tests. The effect of material ductility on the failure of truss-core sandwich panels is revealed. Combining the elasto-plastic deformation under the tracked load and the low cycle fatigue life of aluminum, a feasible method for predicting the fatigue life of joist-core sandwich panels under a tracked load is proposed. Skeji’c et al. [29] obtained a data collection for aluminum products found mainly in the European market for aluminum alloy series 1xxx, 5xxx, 6xxx and 7xxx, mainly for the relevant mechanical properties. They were fitted to distributions and the associated quartiles were determined, and the ratios of nominal to characteristic and design values were also analyzed. The variation in the resulting ratios indicates that the majority of the nominal values are economical and reliable. However, some adjustments to the nominal values are required to achieve a uniform level of reliability in terms of alloy selection and tempering. Simulation analysis is an effective method for the analysis of the forming and static properties and fatigue characteristics of aluminum alloy structures for high-speed trains. Graciano et al. [30] studied the design resistance of extruded aluminum beams subjected to concentrated loads. By comparing the numerically calculated resistance with that calculated using the current design provisions in EC9, it was shown that the EC9 provisions underestimate the resistance. Zhang et al. [31] carried out a computer simulation analysis for a complex hollow aluminum profile-forming process used on high-speed trains. The experimental verification shows that the results of the virtual die test are credible and can provide theoretical guidance for the practical maintenance of complex extrusion dies in the workshop.

As a whole, there are few studies on the strength and fatigue life of underframe suspension structures of high-speed trains in the existing literature. One of the main goals of this study was to carry out the finite element simulation analysis of a single-point suspension structure to obtain the stress distribution and maximum stress level of the structural components and to determine the stress state of the structure. On this basis, eight specimens were cut from the high-speed train floor structure. First, tensile tests were carried out to obtain the load–displacement curves. Second, fatigue performance tests were carried out to obtain fatigue performance curves of real high-speed train suspension structures and probabilistic fatigue performance curves with given confidence and reliability levels, which can be used to guide the actual design and probabilistic life assessment of the structures.

## 2. Structure and Materials

**Figure 1:**
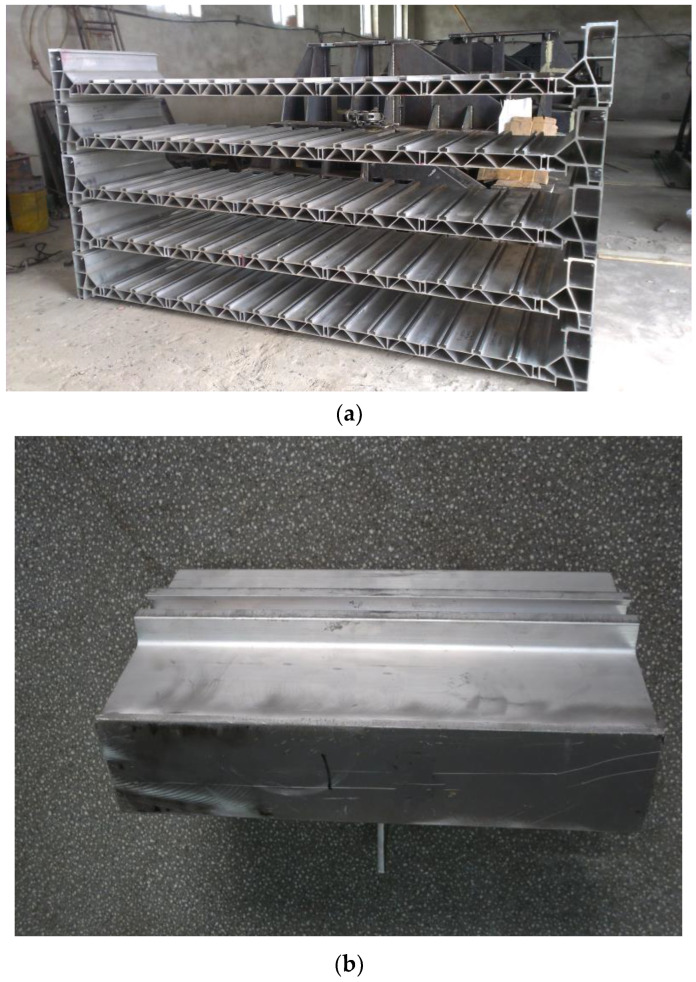
Real structures and test structural component. (**a**) Real structures; (**b**) test structural component cut from real structures.

**Figure 2:**
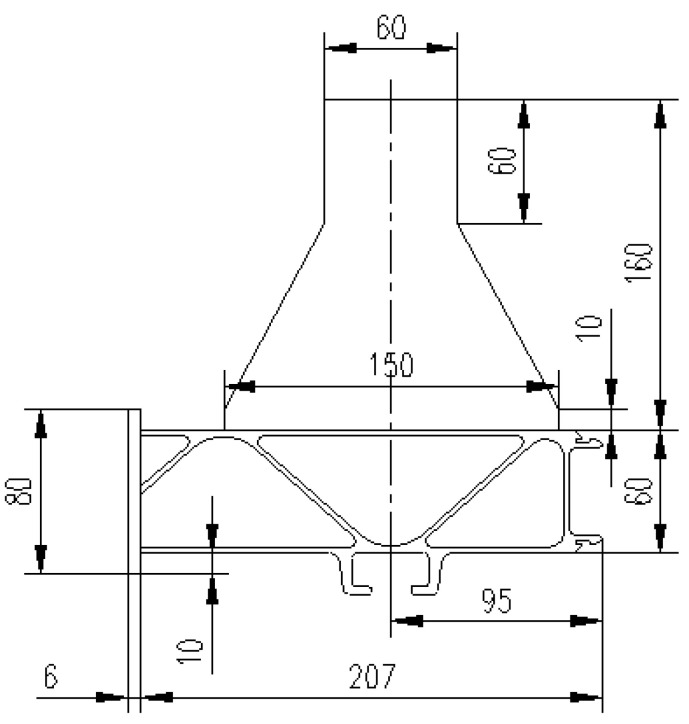
Engineering drawings of the test structural component (all dimensions are in mm).

**Figure 3:**
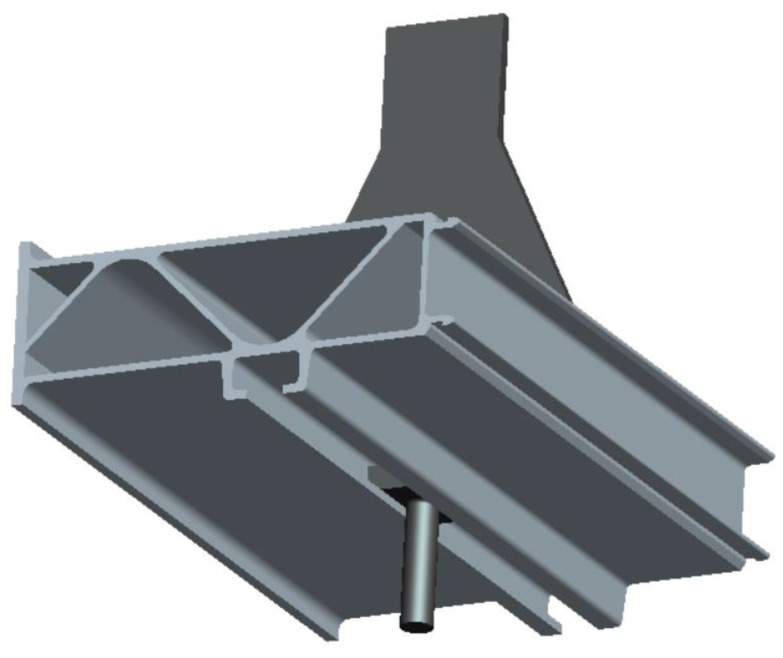
Three-dimensional solid model of the suspension structure.

**Figure 4:**
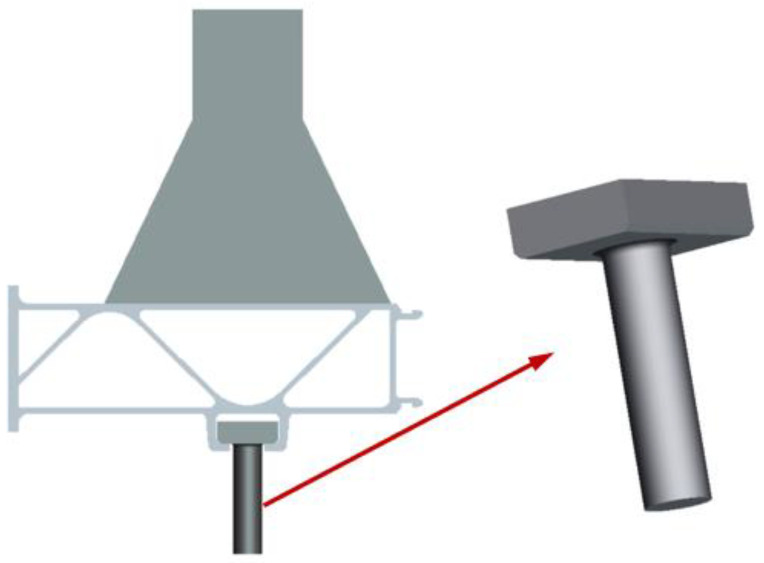
Suspension structure and connecting bolt.

The test structural components were parts of the structure taken from a real high-speed train floor structure, as shown in Figure 1. To facilitate the test, a load-bearing section was welded to the upper part. The engineering diagram of the structural component is shown in Figure 2, and the surface of the structural component remained unchanged. The material was an extruded aluminum alloy profile, material grade 6005A-T6. The mechanical properties and chemical composition of the material are shown in Table 1 and Table 2. The equipment of the train was suspended at the bottom of the train, the specific suspension structure was a slide groove and bolt structure, and the bolt was a square head bolt. The 3D solid model of the base plate suspension structure is shown in Figure 3 and the specific suspension connection structure is shown in Figure 4.

## 3. Simulation Analysis

**Figure 5:**
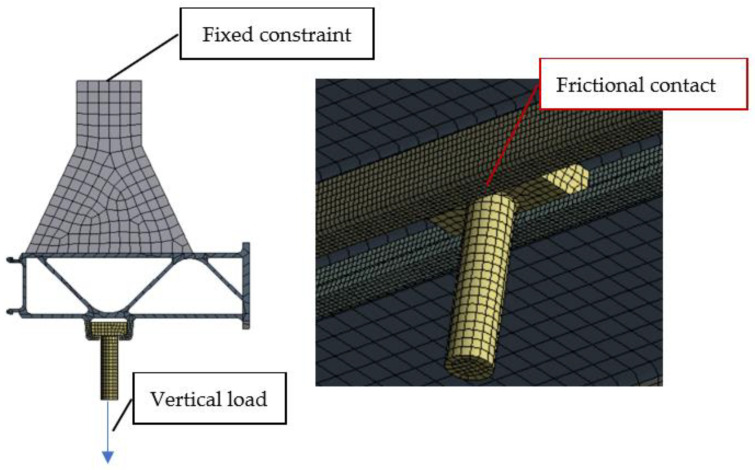
Finite element model of the test structure.

As the stress concentration exists in the connection structure, the actual stress in its local area will be much larger than the nominal stress, so a finite element model of the test member and bolt was established in this work to simulate and analyze it. The finite element model of the test structure is shown in Figure 5. The ambient conditions for the analysis were room temperature and one standard atmosphere. The overall structure uses hexahedral elements, which is conducive to controlling the size and number of meshes. Due to a certain stress concentration at the rounded corners of the lower part of the slot, some mesh refinement was carried out at this location. The refinement mesh size was 2 mm. To ensure convergence of the mesh, we achieved this by gradually reducing the mesh size. When the stress distribution and stress levels do not change significantly as the mesh size is continuously reduced, we can assume that the current mesh size is appropriate.

A fixed restraint was applied to the clamping part at the top of the structure and an axial load of 11.0kN was applied to the bottom surface of the bolt. The contact between the square-headed bolt and the slot was frictional contact with a friction coefficient of 0.3, and the material of the bolt was carbon structural steel.

Since the stress state of the entire structural component was in the elastic range, a linear elastic model was chosen for the material model. As this was a static analysis, the process was relatively simple and most of the solution parameters did not need to be set specifically.

**Figure 6:**
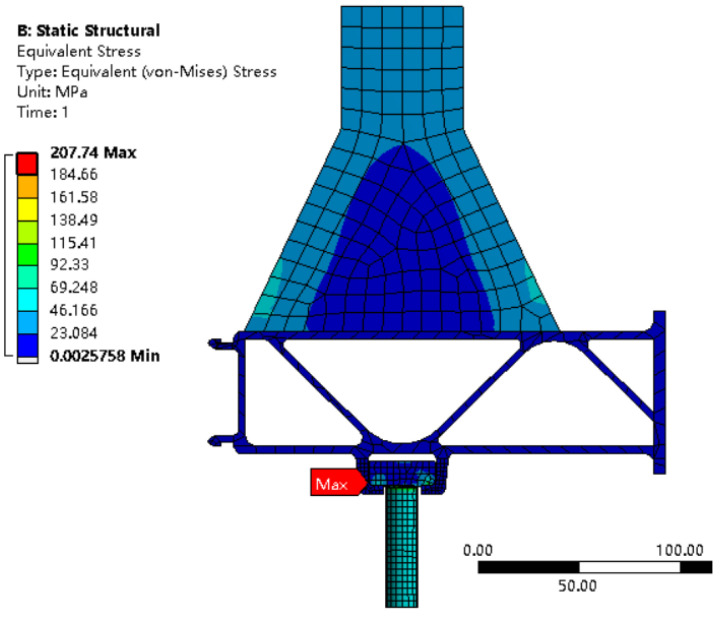
Overall stress distribution diagram.

**Figure 7:**
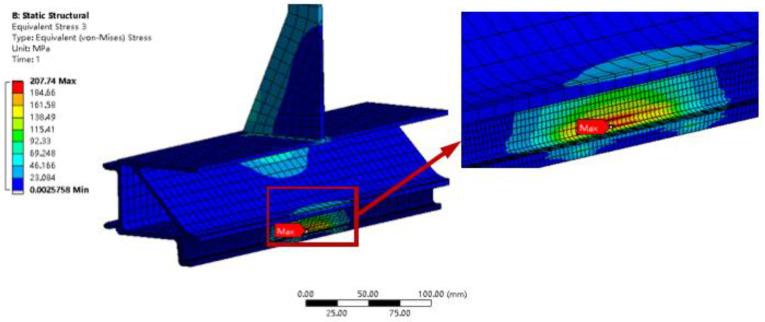
Location of the local maximum stress point.

Figure 6 shows a contour diagram of the stress distribution of the simulation model, from which it can be seen that the location of the maximum stress is at the transition fillet of the slide slot. Figure 7 shows the location of the local maximum stress point and the local enlargement, from which the specific location of the maximum stress point can be seen, with a maximum stress value of 207.74 MPa.

## 4. Experimental Procedure

In order to clarify the static mechanical performance and fatigue performance of the structural members, the static tensile test and fatigue test of the structural members were carried out, respectively, and the tests were completed on the electro-hydraulic servo fatigue testing machine.

### 4.1. Static Tensile Test

**Figure 8:**
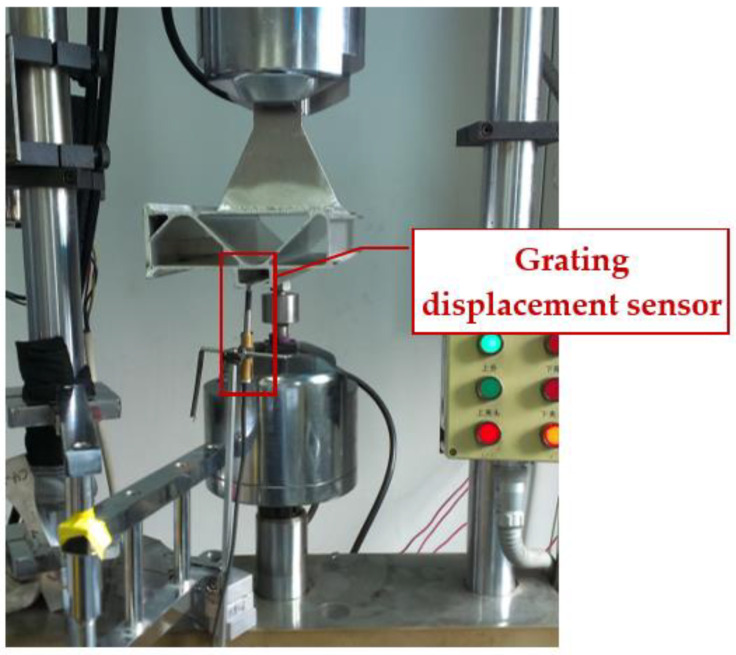
Test structure component and loading system.

Determining the load–displacement relationship of structural members is important for the life and strength assessment of the structure. In order to determine the load–displacement relationship of the floor chute suspension test piece and the strength of damage, the tensile test of the floor chute suspension test piece was carried out. The ambient conditions for the test were room temperature and one standard atmosphere. During the tensile process, the load values and the corresponding displacement values were recorded in real time. The displacement measurement was achieved by an electro-hydraulic servo fatigue tester. When the chuck of the fatigue tester moves, the displacement sensor inside the chuck will measure the displacement and reflect it to the interface of the control software. Figure 8 is an image of the load–displacement measurement test; the test is a displacement-controlled tensile test, the loading rate is 2 mm/min, the load value and the corresponding displacement value are recorded every minute, and the test is terminated when the specimen is fractured or the bolt connection is detached. In addition, we also applied a grating displacement sensor as an auxiliary measurement means for parallel measurements, with a grating sensor measurement accuracy of 0.001 mm.

### 4.2. Fatigue Performance Test

**Figure 9:**
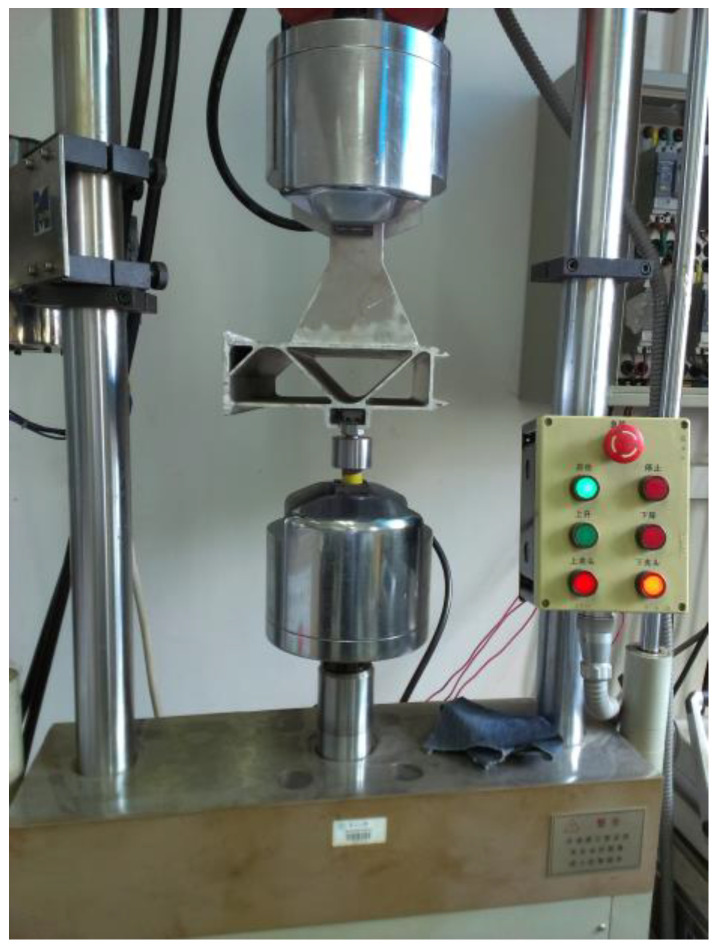
Test structure component being clamped on the tester.

The fatigue test was carried out with a servo-hydraulic fatigue tester. The upper and lower chucks were hydraulically driven, with automatic centering and clamping force adjustment functions. In order to obtain the fatigue performance of aluminum alloy structural parts, the test method of axial force control was used. The ambient conditions for the test were room temperature and one standard atmosphere. The test loads and constraints were as follows: the minimum load was 8.0 kN, the maximum load was 15.0 kN, the load increment was around 2 kN, the stress ratio was 0.54, and the test frequency was 10 Hz. The total number of specimens was 8, as the test structural members were cut from the real high-speed train floor. Figure 9 shows the structural component after clamping on the test machine. Displacement measurement was performed by displacement sensors.

## 5. Results and Discussion

**Figure 10:**
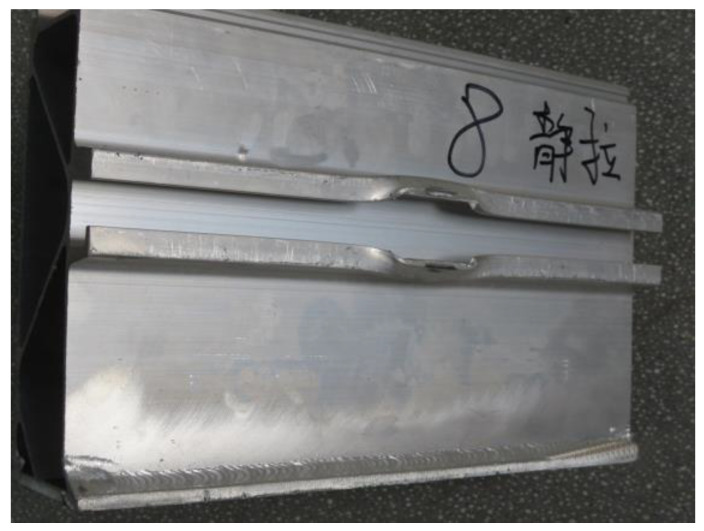
Failed test structure component in tensile test.

**Figure 11:**
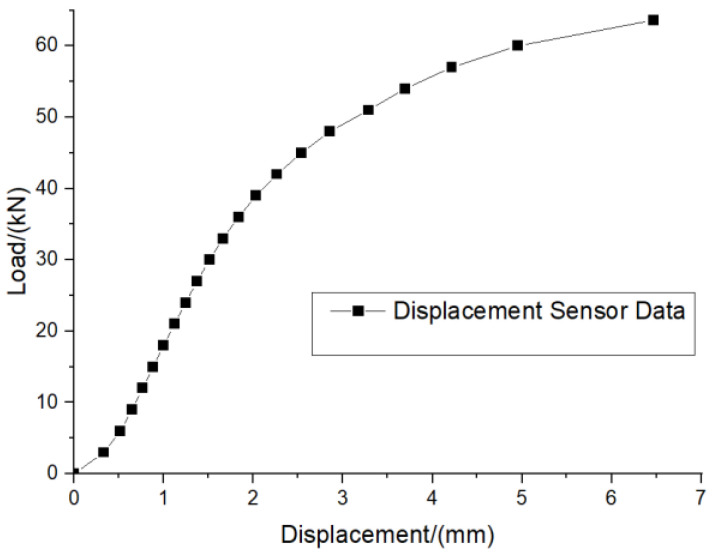
Load–displacement curve.

Figure 10 shows the failure structure of the test piece after the static tensile test. Figure 11 shows the load–displacement relationship curve of the structural member. It can be seen from Figure 11 that when the loading level is less than 35 kN, the load–displacement relationship is roughly linear, and the static tensile fracture limit load of the structural member is between 60 and 65 kN.

**Figure 12:**
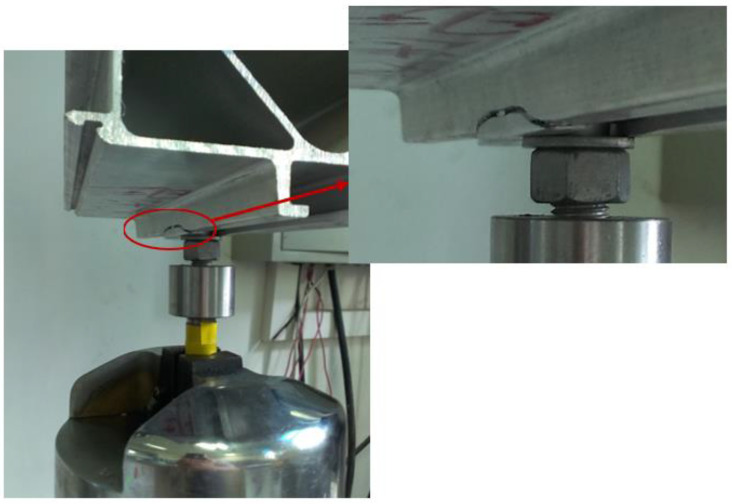
Location of a fatigue fracture in the structural component.

Figure 12 shows an image of the fatigue test after it was stopped. As can be seen from the figure, a fracture occurred at the corner of the chute rounding of the structural member, and the location of the fracture was consistent with the location of the maximum stress value analyzed in the finite element simulation. When the tensile test was carried out, the large plastic deformation of the structural member occurred, which is also consistent with the results of the finite element simulation analysis. This indicates that the stress results obtained through the finite element analysis are accurate.

**Figure 13:**
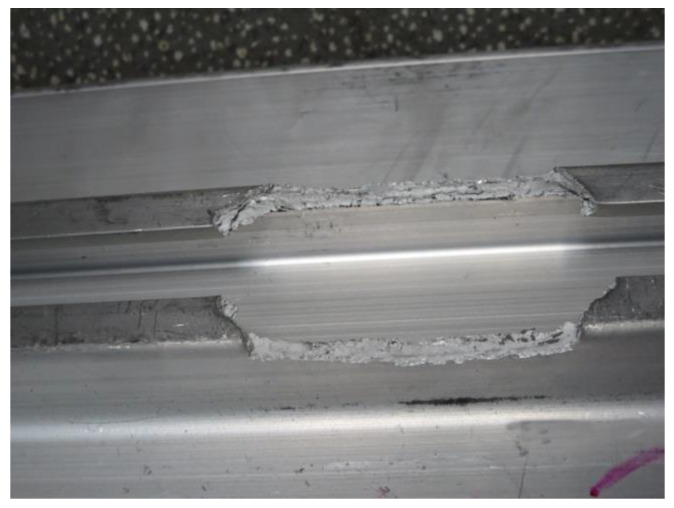
Fatigue fracture profile of a structural component.

Figure 13 shows the fracture profile of a fractured test structural member. It can be seen that the fracture has a brittle fracture feature, with both sides of the chute completely falling off the base.

Usually, the fatigue life data follow a log-normal distribution, and the eight data obtained from the test are fitted to a straight line in a double logarithmic coordinate system. According to the power function formula, there are

(1) $$S^{m}\;N\;=\;C$$

where m and C are the material constants.

Taking the logarithm of both sides of the above equation, we have mlogS + logN = logC. Collating this equation, we have

(2) $$\mathrm{logS}=\frac{1}{m}\mathrm{logC}-\frac{1}{m}$$

**Figure 14:**
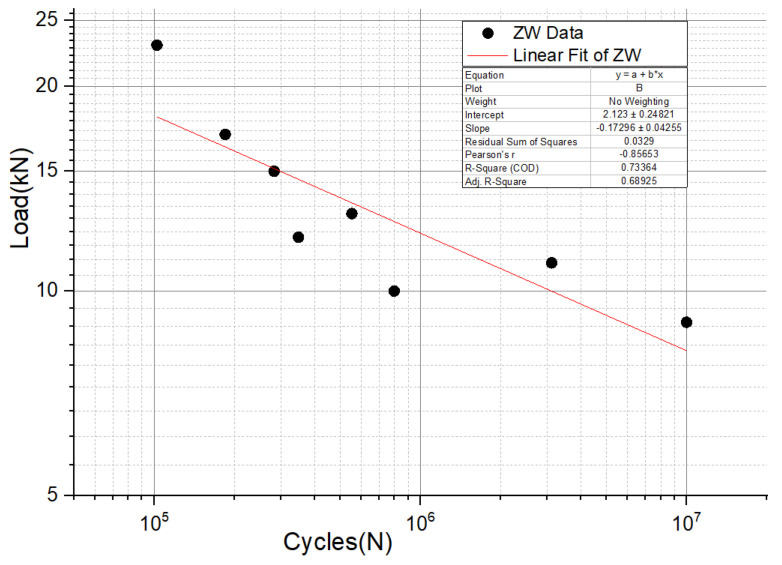
Fatigue life data and load–life curve of structure component.

For floor slide-hanging structural elements, the fitted curve in Figure 14 has the form y = a + bx, the slope of the material load–life curve (S-N curve) is −0.173, and the intercept is 2.123.

Using Equation (2), we can obtain that when the load level is 11 kN, the corresponding fatigue life is 1,823,055 cycles.

For the design analysis of suspension structural members, load–life curves based on different confidence levels and different reliability levels (probabilistic life curves, P-S-N curves) are the basis, and this study obtained design curves under different confidence levels and different reliability levels based on the one-side tolerance factor method [32,33]. This was achieved by translating the median S-N curve to the left by a certain number of units and was calculated as follows [34,35].

(3) $$X_{L}={\overset{—}{X}}_{L}\;-\;K\;\times \;S$$

where ${\overset{—}{X}}_{L}$ is the median lifetime, X_L_ is the lifetime at a given confidence and reliability level, K is the unilateral tolerance coefficient, and S is the standard deviation of the lifetime of the specimens.

(4) $$K=-\frac{t_{\gamma }(n-1,-\sqrt{n\mu_{R}})}{\sqrt{n}}$$

where $t_{\gamma }(n-1,-\sqrt{n\mu_{R}})$ is the γ-quantile of the non-central t-distribution, and $\mu_{R}$ is the standard normal bias of the reliability.

**Figure 15:**
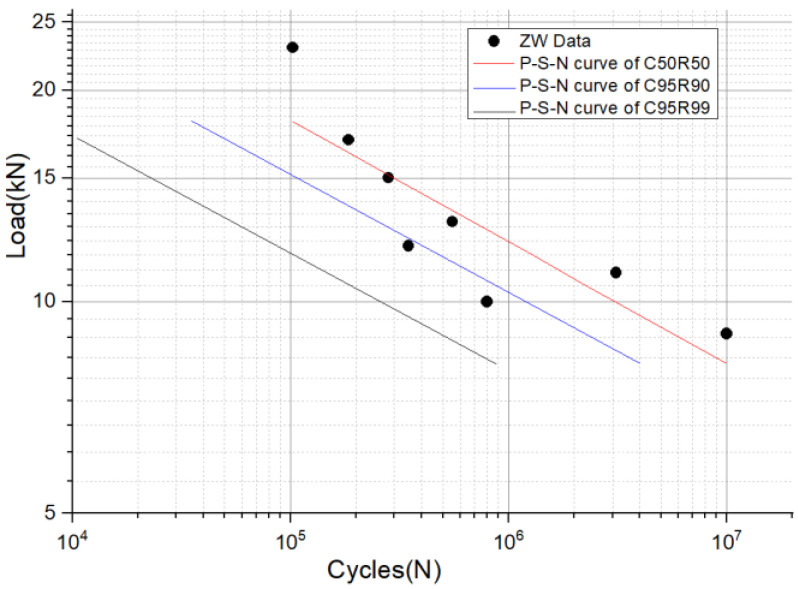
Load–life curve and probability load–life curves.

Based on the above equations, we can obtain the probabilistic fatigue life curves of the structural members. Figure 15 shows the load–life curve and probability load–life curves (P-S-N curves) of fatigue data with different confidence levels and reliability.

## 6. Conclusions

Due to the requirements of energy saving and environmental protection, aluminum alloy structural parts are used in large numbers in high-speed trains, but the performance of aluminum alloy is not inferior to that of traditional steel. Therefore, its tensile and fatigue properties need to be analyzed and verified. In this paper, the tensile and fatigue properties of aluminum alloy suspension structural members used for suspension components have been simulated and experimentally studied, and the following conclusions have been obtained.

1. The maximum stress site of the suspension structure is at the transition round corner of the suspension slot, where there is a stress concentration. When the load level is 11.0 kN, the maximum stress value is 207.74 MPa.
2. The tensile test shows that under a large load, the hanging slot will undergo large plastic deformation, the ultimate load-bearing level of the suspension slot is 65 kN, and the static strength can meet the needs of equipment suspension.
3. The median load–life curve obtained by fitting the test data shows that when the load level is 11 kN, the corresponding fatigue life is 1,823,055 cycles. This result has practical significance for the rational arrangement of the number of suspension points.
4. The fatigue tests show that the failure site of the structural component is at the transition corner of the suspension slot, which is consistent with the location of the maximum stress from the finite element analysis. The fatigue life of aluminum structures is influenced by many factors and is very sensitive to various defects. As a rule, the fatigue life of structures is highly dispersive. Therefore, a design curve based on a certain level of confidence and reliability, known as a P-S-N curve, should be used when designing the structure, rather than the median S-N curve.

## Figures and Tables

**Table 1:** Material parameters of the structure material.

Material Parameters	Elastic Module (MPa)	Poisson Ratio	Ultimate Tensile Strength (MPa)	Yield Stress (MPa)	Density (t/mm^3^)
Value	70,000	0.32	255	215	2.71E-9

**Table 2:** Chemical composition of the structure material.

Material	Si	Fe	Cu	Mg	Mn	Cr	Zn	Ti	Other	Al
6005A	0.5 ~ 0.9	≤0.35	≤0.30	0.4 ~ 0.7	≤0.50	≤0.30	≤0.20	≤0.10	≤0.15	Balance

## Data Availability

All the data used in this study can be found in the article.
